# Association between high-density lipoprotein cholesterol and all-cause mortality in the general population of northern China

**DOI:** 10.1038/s41598-019-50924-4

**Published:** 2019-10-08

**Authors:** Xintao Li, Bo Guan, Yanjun Wang, Gary Tse, Fuquan Zou, Bin Waleed Khalid, Yunlong Xia, Shouling Wu, Jianhui Sun

**Affiliations:** 1 Department of Cardiology, The First People’s Hospital of Changzhou, Changzhou, 213000 China; 2 Li Ka Shing Institute of Health Sciences, Chinese University of Hong Kong, Hong Kong Special Administrative Region, Hong Kong, 999077 China; 3 Department of Cardiology, First Affiliated Hospital of Dalian Medical University, Dalian, 116011 China; 4 Department of Cardiology, Kailuan General Hospital, Tangshan, 063000 China

**Keywords:** Risk factors, Lifestyle modification

## Abstract

Recent studies proposed reasonable doubts about the good prognosis of very high levels of high-density lipoprotein cholesterol (HDL-c). We aimed to investigate the association between HDL-c levels and all-cause mortality using data from an observational cohort study in northern China from 2006 to 2015. The study population was stratified into six groups by HDL-c levels in mg/dl (<40, 40–49, 50–59, 60–69, 70–79, ≥80). Cox hazards regression models were used to estimate the association between HDL-c levels and all-cause mortality. In total, 100,070 participants (aged 51.9 ± 12.7 years) were included in the current analysis. During a mean follow-up of 8.76 years, 7,362 deaths were identified (mortality rate, 8.40 per 1000 person-years). There was a significant interaction effect between age and HDL-c levels (P for interaction < 0.001). Among individuals aged 65 and older, no significant association was found between HDL-c levels and total mortality. In contrast, HDL-c levels showed a U-shaped relationship with all-cause mortality in younger participants (<65 years old), and very high HDL-c levels (≥80 mg/dl) were independently associated with increased total mortality risk compared with the reference level (60 to 69 mg/dl). These findings suggest that very high HDL-c levels may not represent a good prognosis, especially in younger individuals.

## Introduction

High-density lipoprotein cholesterol (HDL-c), first reported to be inversely associated with ischemic heart disease by Gofman *et al*.^1^, has been widely recognized as an important factor in the development of cardiovascular (CV) mortality and morbidity^2,3^. Numerous studies have established that a low HDL-c level is associated with a high risk of CV events and mortality independent of race, ethnicity, and sex^3–6^. As a consequence, it was assumed that attainment of higher levels of HDL-c would reduce the risk of cardiovascular diseases (CVD) or even extend the lifespan^7^. However, recent randomized clinical trials that raised HDL-c levels through medical intervention failed to find any significant clinical benefit to CVD or mortality^8–12^. Nonetheless, the plausible reasons for the failure of these HDL-c-raising interventions indicated that the HDL-c hypothesis remained controversial^3^.

Limited observational cohort data have suggested a plateau effect or increased risks for CVD and total mortality events in individuals with extremely high HDL-C levels^13–15^. However, due to the small sample size of individuals with very high HDL-c, most studies lack the power to draw robust conclusions about the risks of total mortality across the entire spectrum of HDL-c. Moreover, since HDL-c is known to be closely intertwined with other demographic and lifestyle factors^3^, recent epidemiological and genetic studies suggested that the associations between HDL-c level and all-cause mortality may vary in diverse populations with different environmental and genetic background information^3,16^.

To the best of our knowledge, no large general population-based study has investigated the association between the entire spectrum of HDL-c levels and all-cause mortality in China. The Kailuan Study^17^ in northern China is a large prospective cohort and documents detailed covariate data, including demographics, laboratory values, and cardiovascular risk factors. Thus, the study provides us a unique opportunity to quantify the hazards of all-cause mortality across the full range of HDL-c levels using both unadjusted and adjusted models.

## Methods

### Study design and participants

The Kailuan Study is a prospective cohort based on the Kailuan community in Tangshan city in northern China, which represents the Chinese population from a socioeconomic perspective. Details of the study have been described elsewhere^18^.

Participants at baseline (2006–2007) included 101510 men and women, aged 18 to 98 years, who completed questionnaires by interviews and had clinical examinations that were conducted in the 11 hospitals responsible for the healthcare of this community. These participants were then followed through 2014–2015 with repeated questionnaires and clinical and laboratory examinations every two years.

The inclusion criteria of the current study were (1) 18 years of age or older, (2) participation in the first examination, and (3) signed agreement to participate in this study and informed consent. The exclusion criteria were (1) incomplete information for blood lipids and other relevant covariates, (2) pregnancy during the examination period, and (3) refusal to sign the informed consent. A total of 100070 participants were under current analysis. We then followed these participants from the baseline examination to the data of death or until they were otherwise lost to follow-up. The diagnosis of all-cause death (except accidental) was determined and recorded by state vital statistics offices.

The protocol for this study was in accordance with the guidelines of the Helsinki Declaration and was approved jointly by the Ethics Committee of the Kailuan General Hospital and the First People’s Hospital of Changzhou. All participants gave their written informed consent.

### Determination of blood lipids

Blood samples were collected from the antecubital vein in the morning after an overnight fasting period and transferred into vacuum tubes containing ethylene diamine tetraacetic acid (EDTA) for processing. Detailed information is available elsewhere^19^. Briefly, the samples were centrifuged at 3000 × g for 10 minutes within 4 hours of storage. HDL-c, low-density lipoprotein cholesterol (LDL-c), total cholesterol, and triglycerides were measured by an enzymatic method (Mind Bioengineering Co. Ltd, Shanghai, China; interassay CV: <10%). All blood variables were measured using an autoanalyzer (Hitachi 747; Hitachi, Tokyo, Japan) at the central laboratory of the Kailuan General Hospital.

### Determination of covariates

Demographic data and smoking status, alcohol intake, education level, physical activity and information regarding CVD were collected from questionnaires at baseline in 2006. Detailed procedures on covariate measurements have been described elsewhere^20^. Anthropometric parameters and blood pressure were measured during the interview. Diabetes mellitus was defined based on a fasting blood glucose value ≥7.0 mmol/L or medical treatment for diabetes. Hypertension was defined as systolic blood pressure ≥140 mmHg or diastolic blood pressure ≥90 mmHg or use of antihypertensive medications. Chronic kidney disease (CKD) was defined as the estimated glomerular filtration rate (eGFR) <60 mL/min/1.73 m2, calculated using the CKD-EPI formula. Prevalent CVD were defined as prior physician-diagnosed myocardial infarction and stroke.

### Statistics analysis

To investigate the relationship between full ranges of HDL-c levels and mortality, the individuals were stratified into predetermined groups based on HDL-c levels in mg/dl (<40, 40 to 49, 50 to 59, 60 to 69, 70 to 79, and ≥80). Continuous data were expressed as the mean ± standard deviation and compared using one-way ANOVA or nonparametric tests. Categorical variables were described by percentages and compared using the chi-square test.

Survival curves were calculated using the Kaplan-Meier method, and the differences in survival probability between HDL-c levels were compared using the log-rank test. Cox proportional hazard models were used to calculate the survival models by hazard ratios and 95% CIs after verifying the proportional hazards assumption with Schoenfeld residuals. Reference groups were the strata containing the levels of HDL-c associated with the lowest mortality rates. The association between HDL-c levels and all-cause mortality was tested by an unadjusted model initially and then adjusted for potential confounders, including age, sex, education, physical activity, smoking, drinking, body mass index, non-HDL-c, triglycerides, hypertension, diabetes mellitus, CKD, and history of myocardial infarction, stroke, and malignancy.

Interactions between HLD-c levels and age, sex, BMI, and LDL-c levels were tested by adding the cross product of two variables into the main effects model. A significant interaction effect on all-cause mortality was identified between HDL-c levels and age, but not for other factors. Thus, the data were stratified by age and analyzed separately for the elderly (≥65) and younger (<65) participants^21^.

To avoid the potential bias resulting from occult diseases at baseline, individuals were subsequently stratified by hypertension, diabetes mellitus, CKD, and CVD. In addition, sensitivity analyses were conducted by excluding individuals with malignancy and CVD at baseline, lipid-lowering medication users, and participants who died in the first year of follow-up.

A P value < 0.05 was considered statistically significant, and all statistical tests for significance were 2-tailed. Statistical analyses were performed using SAS software, version 9.1 (SAS Institute, Cary, North Carolina, USA) and SPSS 13.0 (SPSS Inc, Chicago, IL).

## Results

### Baseline characteristics

General characteristics of the study population by HDL-c level strata are presented in Table 1. The mean age of the participants was 51.91 years, and 79.9% were men. The mean HDL-c level was 59.73 mg/dl, the mean non-HDL-c level was 131.27 mg/dl, and the mean triglyceride level was 112.39 mg/dl. Older participants, women, and those who had a higher daily alcohol consumption and higher blood pressure were more likely to have a high level of HDL-c. Conversely, individuals with lower HDL-c levels had comparatively higher BMI, estimated glomerular filtration rate, non-HDL-c level, and prevalence of stroke and myocardial infarction. There was no consistent relationship between LDL-c and HDL-c levels, whereas total cholesterol was positively and linearly related to HDL-c levels.

**Table 1:** Subject baseline characteristics.

Characteristics	Levels of HDL cholesterol					
	<40 mg/dl n = (6674)	40–50 mg/dl n = (19999)	50–60 mg/dl n = (29204)	60–70 mg/dl n = (22975)	70–80 mg/dl n = (12493)	≥80 mg/dl n = (8725)
Age (years)	51.31 ± 12.90	50.91 ± 13.08	51.23 ± 12.72	51.54 ± 12.50	53.31 ± 12.22	55.87 ± 11.52
Male, n (%)	5470(82.0)	16817(84.1)	23602(80.8)	17848(77.7)	9593(76.8)	6583(75.4)
BMI (kg/m2)	25.46 ± 3.60	25.50 ± 3.47	25.24 ± 3.43	24.84 ± 3.47	24.52 ± 3.47	24.30 ± 3.54
Completed high school, n (%)	1314(19.7)	4616(23.1)	6152(21.1)	4291(18.7)	1935(15.5)	1116(12.8)
Daily smoker, n (%)	2019(30.3)	6784(33.9)	8948(30.6)	6323(27.5)	3493(28.0)	2475(28.4)
Daily alcohol user, n (%)	860(12.9)	3123(15.6)	4929(16.9)	4043(17.6)	2532(20.3)	1971(22.6)
Regular exercise, n (%)	1276(19.1)	3448(17.2)	4409(15.1)	2973(12.9)	1735(13.9)	1315(15.1)
Systolic BP, mmHg	129.54 ± 20.23	129.89 ± 20.57	130.57 ± 20.97	130.90 ± 21.15	132.45 ± 21.49	135.10 ± 21.73
Diastolic BP, mmHg	82.76 ± 11.65	83.24 ± 11.75	83.43 ± 11.72	83.40 ± 11.77	83.81 ± 11.83	84.57 ± 12.00
FBG, mmol/L	5.43 ± 1.73	5.52 ± 1.62	5.50 ± 1.62	5.44 ± 1.64	5.43 ± 1.70	5.52 ± 2.01
hs-CRP, mg/L	0.89 (0.34,2.20)	0.90 (0.36,2.30)	0.85 (0.31,2.30)	0.76 (0.30,2.10)	0.72 (0.26,2.05)	0.79 (0.27,2.47)
TC, mg/dl	170.82 ± 40.03	181.53 ± 38.46	188.72 ± 42.67	192.95 ± 43.96	201.21 ± 44.34	216.05 ± 48.70
LDL-C, mg/dl	84.19 ± 34.18	93.25 ± 34.81	93.20 ± 35.11	90.29 ± 34.29	87.94 ± 34.52	85.83 ± 39.02
Non-HDL-C, mg/dl	136.33 ± 40.42	135.79 ± 38.32	133.65 ± 42.67	128.21 ± 43.94	126.82 ± 44.18	123.56 ± 49.97
TG, mg/dl	118.58 (77.88,191.15)	120.35 (83.19,179.65)	114.16 (80.53,171.68)	108.85 (77.88,161.06)	107.08 (76.99,161.95)	109.73 (76.11,167.26)
eGFR, mL/min/1.73 m2	84.23 ± 29.52	84.20 ± 24.58	82.27 ± 25.34	80.32 ± 24.01	80.50 ± 25.15	80.79 ± 29.42
Hypertension, n (%)	2658(39.8)	8352(41.8)	12594(43.1)	10103(44.0)	5736(45.9)	4428(50.8)
Diabetes mellitus, n (%)	632(9.5)	1904(9.5)	2622(9.0)	1928(8.4)	1115(8.9)	906(10.4)
History of stroke, n (%)	207(3.1)	585(2.9)	781(2.7)	495(2.2)	280(2.2)	190(2.2)
History of MI, n (%)	102(1.5)	274(1.4)	395(1.4)	278(1.2)	141(1.1)	114(1.3)
History of malignancy, n (%)	26(0.4)	69(0.3)	99(0.3)	89(0.4)	48(0.4)	45(0.5)
Antihypertensive drugs, n (%)	860(12.9)	2537(12.7)	3397(11.6)	2210(9.6)	1253(10.0)	964(11.0)
Lipid-lowering medication, n (%)	70(1.0)	220(1.1)	267(0.9)	190(0.8)	121(1.0)	90(1.0)
Mortality, n (%)	499(7.5)	1416(7.1)	2084(7.1)	1570(6.8)	965(7.7)	828(9.5)

Abbreviations: BMI, body mass index; FBG, fasting blood glucose; hs-CRP, high sensitivity C-reactive protein; TC, total cholesterol; LDL-C, low-density lipoprotein cholesterol; TC, total cholesterol; TG, triglycerides; MI, myocardial infarction.

### 3.2 Mortality and HDL-c levels

During a mean follow-up of 8.76 years, 7,362 deaths from various causes were identified among the 100,070 participants. The overall mortality was 8.40 (95% CI 8.21 to 8.59) per 1000 person-years. There was a significant interactive effect between age and HDL-c levels on all-cause mortality (P for interaction <0 0.001). In contrast, no significant modifying effects of sex and other factors with HDL-c levels were found. Table 2 shows the mortality rate across HDL-c levels in younger (<65) and older (≥65) participants separately. Individuals at the HDL-c level ranging from 60 to 69 mg/dl and in the highest HDL-c stratum (≥80 mg/dl) had the lowest overall mortality rate of the younger and older participants, respectively. The survival curves of younger and older individuals according to HDL-c levels are presented in Supplemental Fig. 1A,B.

**Table 2:** Association between HDL-c levels and all-cause mortality stratified by age groups.

Age < 65	Levels of HDL Cholesterol					
	<40 mg/dl	40–50 mg/dl	50–60 mg/dl	60–70 mg/dl	70–80 mg/dl	≥80 mg/dl
Death	243	669	984	716	425	367
Person-years	50511	151925	221432	174298	91652	60081
Mortality (per 1000)	4.81	4.40	4.44	4.11	4.64	6.11
Model 1	1.16(1.01–1.35)*	1.07(0.96–1.19)	1.08(0.98–1.19)	1.00(ref)	1.13(1.00–1.27)*	1.49(1.32–1.69)***
Model 2	1.13(0.98–1.31)	1.06(0.95–1.18)	1.08(0.98–1.18)	1.00(ref)	1.06(0.94–1.20)	1.28(1.12–1.45)***
Model 3	1.11(0.96–1.29)	1.05(0.94–1.17)	1.09(0.99–1.20)	1.00(ref)	1.05(0.93–1.18)	1.28(1.12–1.45)***
Model 4 (Overall)	1.11(0.96–1.29)	1.05(0.95–1.17)	1.09(0.98–1.20)	1.00(ref)	1.05(0.93–1.18)	1.27(1.11–1.44)***
Male	1.12(0.96–1.31)	1.06(0.95–1.19)	1.11(1.00–1.23)*	1.00(ref)	1.10(0.96–1.25)	1.30(1.13–1.49)***
Female	1.11(0.68–1.83)	1.04(0.72–1.50)	0.88(0.64–1.21)	1.00(ref)	0.69(0.46–1.04)	1.02(0.70–1.45)
**Age ≥ 65**	**Levels of HDL Cholesterol**					
	**<40 mg/dl**	**40–50 mg/dl**	**50–60 mg/dl**	**60–70 mg/dl**	**70–80 mg/dl**	**≥80 mg/dl**
Death	256	747	1100	854	540	461
Person-years	7532	23195	34324	27165	17969	16067
Mortality (per 1000)	33.99	32.21	32.05	31.44	30.52	28.69
Model 1	1.20(1.03–1.39)*	1.13(1.01–1.27)*	1.12(1.01–1.25)*	1.10(0.98–1.23)	1.05(0.93–1.19)	1.00(ref)
Model 2	1.08(0.92–1.26)	1.03(0.92–1.16)	1.04(0.93–1.16)	1.03(0.92–1.15)	1.01(0.89–1.14)	1.00(ref)
Model 3	1.14(0.97–1.33)	1.10(0.97–1.24)	1.08(0.96–1.21)	1.05(0.94–1.18)	1.00(0.88–1.14)	1.00(ref)
Model 4 (Overall)	1.10(0.94–1.30)	1.05(0.93–1.91)	1.05(0.93–1.18)	1.04(0.92–1.17)	1.00(0.87–1.13)	1.00(ref)
Male	1.08(0.91–1.28)	1.04(0.91–1.18)	1.03(0.92–1.17)	1.03(0.91–1.17)	0.99(0.87–1.14)	1.00(ref)
Female	1.48(0.78–2.82)	1.15(0.72–1.84)	1.21(0.80–1.81)	0.96(0.63–1.46)	0.95(0.62–1.45)	1.00(ref)

*p < 0.05 **p < 0.01 ***p < 0.001.

Model 1: unadjusted.

Model 2: adjusted for age, sex.

Model 3: adjusted for age, sex, education, physical activity, smoking, and drinking.

Model 4: adjusted for age, sex, education, physical activity, smoking, drinking, BMI, hs-CRP, non-HDL-C, triglycerides, chronic kidney disease, diabetes, hypertension, history of MI, history of stroke, history of malignancy.

Abbreviations: BMI, body mass index; FBG, fasting blood glucose; hs-CRP, high sensitivity C-reactive protein; MI, myocardial infarction.

The unadjusted and multivariable adjusted hazard ratios of all-cause mortality in younger and older subjects are shown in Table 2. The HDL-c stratum with the lowest mortality rate was set as the reference group (60 to 69 mg/dl for younger individuals and ≥80 mg/dl for older participants). In the younger group, the lowest stratum (<40 mg/dl) (HR: 1.16, 95% CI: 1.01 to 1.35) and highest 2 strata (70–79 mg/dl and ≥80 mg/dl) of HDL-c levels (HR: 1.13 and 1.49; 95% CI: 1.00 to 1.27 and 1.32 to 1.69) were associated with a high risk for total mortality compared with the reference group (Table 2). The increased mortality risk of the highest level (≥80 mg/dl) remained significant after multivariable adjustment. For the older group, the lowest category of HDL-c (<40 mg/dl) had increased risks for total mortality (HR 1.20; 95% CI 1.03 to 1.39) compared with the highest stratum (≥80 mg/dl). However, the association became nonsignificant when further adjusted for age and other covariates (Table 2).

**Figure 1:**
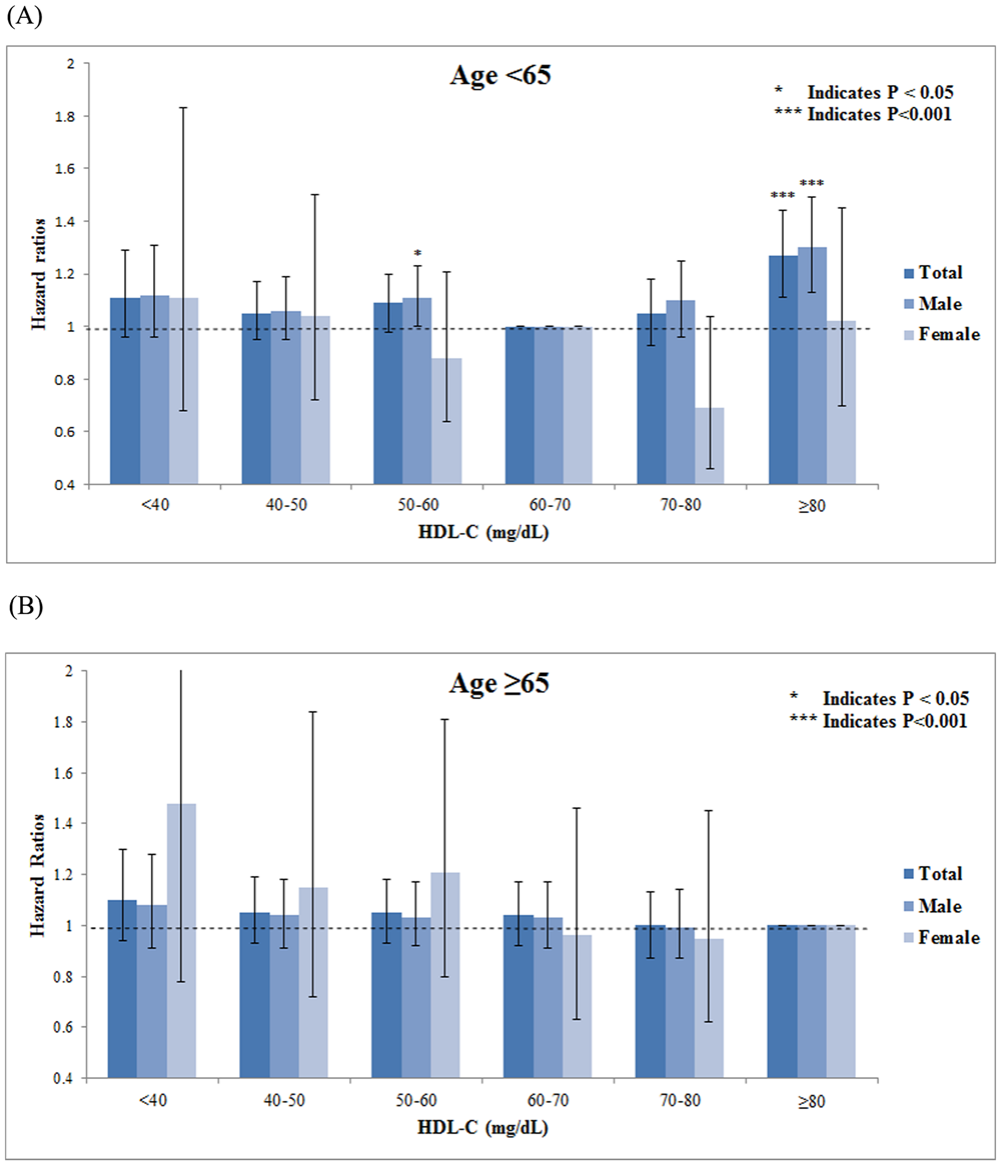
Adjusted hazard ratios of HDL-c levels for all-cause mortality stratified by age groups.

The analyses were repeated stratifying by sex (Fig. 1A,B) and chronic diseases, including hypertension, diabetes mellitus, CKD, and CVD (Table 3). Among younger participants, similar results were found between various subgroups except in female and CKD participants (Fig. 1A and Table 3). The level of lowest risk of total mortality in females was 70–80 mg/dl. In older participants, the inverse linear trend became more apparent in individuals without hypertension and diabetes and in those with CKD (Table 3). The second highest stratum of HDL-c levels (70–79 mg/dl) had lowest overall mortality rates among the elderly participants with CVD, and the association was marginally significant.

**Table 3:** HDL-c levels and multivariable adjusted mortality risk stratified by chronic diseases.

Age < 65	Levels of HDL Cholesterol					
	<40 mg/dl	40–50 mg/dl	50–60 mg/dl	60–70 mg/dl	70–80 mg/dl	≥80 mg/dl
HPT	1.06(0.87–1.29)	1.03(0.89–1.18)	1.09(0.96–1.24)	1.00(ref)	1.00(0.85–1.17)	1.21(1.02–1.43)*
Non-HPT	1.19(0.94–1.49)	1.09(0.92–1.30)	1.08(0.92–1.26)	1.00(ref)	1.12(0.93–1.37)	1.37(1.11–1.68)**
DM	1.16(0.81–1.67)	1.26(0.97–1.64)	1.28(1.01–1.63)	1.00(ref)	1.09(0.81–1.48)	1.44(1.07–1.96)*
Non-DM	1.11(0.94–1.31)	1.01(0.90–1.14)	1.05(0.94–1.17)	1.00(ref)	1.05(0.92–1.20)	1.24(1.07–1.43)**
CKD	0.93(0.60–1.45)	1.18(0.89–1.57)	1.14(0.90–1.45)	1.00(ref)	0.98(0.73–1.31)	1.06(0.77–1.46)
Non-CKD	1.13(0.96–1.33)	1.04(0.92–1.17)	1.08(0.97–1.20)	1.00(ref)	1.06(0.93–1.22)	1.31(1.14–1.51)***
CVD	1.34(0.83–2.15)	1.49(1.04–2.14)	1.37(0.97–1.94)	1.00(ref)	1.17(0.74–1.85)	1.28(0.77–2.15)
Non-CVD	1.09(0.93–1.28)	1.01(0.90–1.13)	1.06(0.96–1.17)	1.00(ref)	1.04(0.91–1.18)	1.27(1.11–1.45)***
**Age** **≥ 65**	**Levels of HDL Cholesterol**					
	**<40 mg/dl**	**40–50 mg/dl**	**50–60 mg/dl**	**60–70 mg/dl**	**70–80 mg/dl**	**≥80 mg/dl**
HPT	1.01(0.83–1.24)	0.99(0.85–1.15)	1.01(0.88–1.16)	1.00(0.86–1.15)	0.94(0.80–1.10)	1.00(ref)
Non-HPT	1.32(0.99–1.74)	1.20(0.96–1.50)	1.13(0.92–1.40)	1.13(0.91–1.39)	1.10(0.88–1.38)	1.00(ref)
DM	0.99(0.67–1.45)	1.00(0.74–1.34)	1.10(0.83–1.46)	1.02(0.76–1.36)	0.94(0.68–1.29)	1.00(ref)
Non-DM	1.13(0.95–1.35)	1.06(0.92–1.22)	1.04(0.92–1.18)	1.04(0.91–1.18)	1.01(0.88–1.16)	1.00(ref)
CKD	1.21(0.92–1.60)	1.13(0.91–1.39)	1.01(0.83–1.24)	1.12(0.92–1.37)	1.04(0.83–1.29)	1.00(ref)
Non-CKD	1.04(0.85–1.28)	1.02(0.87–1.18)	1.07(0.93–1.23)	0.99(0.86–1.15)	0.99(0.84–1.16)	1.00(ref)
CVD	1.07(0.72–1.59)	0.84(0.60–1.17)	0.93(0.68–1.27)	0.93(0.67–1.29)	0.68(0.46–1.00)	1.00(ref)
Non-CVD	1.09(0.91–1.31)	1.09(0.95–1.24)	1.06(0.94–1.20)	1.05(0.92–1.19)	1.04(0.91–1.20)	1.00(ref)

*p < 0.05 **p < 0.01 ***p < 0.001.

Model adjusted for age, sex, education, physical activity, smoking, drinking, BMI, hs-CRP, non-HDL-C, triglycerides, chronic kidney disease, diabetes, hypertension, history of MI, history of stroke, history of malignancy.

Abbreviations: BMI, body mass index; FBG, fasting blood glucose; hs-CRP, high sensitivity C-reactive protein; MI, myocardial infarction; HPT, hypertension; DM, diabetes mellitus; CKD, chronic kidney disease; CVD, cardiovascular diseases.

### Sensitivity analysis

To avoid the possibility that these results were driven by potential confounders instead of HDL-c levels, individuals with a history of malignancy and CVD at baseline and those took lipid-lowering medication were excluded separately (Table 4). Sensitivity analyses were also conducted by excluding deaths that occurred during the first year of follow-up. As a result, the association between very high level of HDL-c and all-cause mortality in younger individuals and the modifying effect of age on elderly participants remained unchanged.

**Table 4:** Sensitivity analysis of HDL-c levels and all-cause mortality risk.

Age <65	Levels of HDL Cholesterol					
	<40 mg/dl	40–50 mg/dl	50–60 mg/dl	60–70 mg/dl	70–80 mg/dl	≥80 mg/dl
Model 1	1.11(0.95–1.29)	1.06(0.95–1.18)	1.08(0.98–1.19)	1(ref)	1.06(0.93–1.20)	1.27(1.11–1.44)***
Model 2	1.09(0.93–1.28)	1.01(0.90–1.13)	1.06(0.96–1.18)	1(ref)	1.04(0.91–1.18)	1.27(1.11–1.45)***
Model 3	1.13(0.97–1.31)	1.04(0.93–1.16)	1.09(0.99–1.21)	1(ref)	1.05(0.92–1.18)	1.28(1.12–1.45)***
Model 4	1.09(0.93–1.27)	1.05(0.94–1.17)	1.08(0.98–1.20)	1(ref)	1.03(0.91–1.16)	1.26(1.11–1.44)***
**Age **≥ **65**						
Model 1	1.11(0.94–1.30)	1.06(0.93–1.20)	1.05(0.93–1.18)	1.04(0.92–1.17)	0.99(0.87–1.13)	1(ref)
Model 2	1.09(0.91–1.31)	1.09(0.95–1.24)	1.06(0.94–1.20)	1.05(0.92–1.19)	1.04(0.91–1.20)	1(ref)
Model 3	1.11(0.94–1.31)	1.06(0.93–1.20)	1.05(0.94–1.18)	1.05(0.93–1.18)	1.01(0.89–1.15)	1(ref)
Model 4	1.08(0.91–1.28)	1.07(0.94–1.19)	1.06(0.94–1.19)	1.04(0.92–1.17)	1.00(0.88–1.14)	1(ref)

Model 1 Multivariable adjusted and further excluded malignancy at baseline.

Model 2 Multivariable adjusted and further excluded cardiovascular diseases at baseline.

Model 3 Multivariable adjusted and further excluded lipid-lowering drugs at baseline.

Model 4 Multivariable adjusted and further excluded death within one year.

## Discussion

In this large cohort study including 100,070 unselected individuals, the association between HDL-c levels and all-cause mortality was varied in different age groups, as no significant association was found in elderly participants and a likely U-shaped association existed in individuals younger than 65. These associations were independent of lifestyle factors, clinical factors, and chronic diseases.

Low levels of HDL-c are associated with the development of cardiovascular mortality^2,3,22^. Notably, recent evidence has shown that low HDL-c levels also increase cancer and non-CV/cancer mortality risk^4^. However, when focused on elderly individuals, the association between HDL-c levels and total mortality becomes controversial^23,24^. Our results found that lower HDL-c levels were related to high all-cause mortality in elderly participants. However, these associations became nonsignificant when further adjusted for age and other confounders. Notably, as age increases, it becomes more important in the prediction of mortality, which may diminish the predictive value of other risk factors^25^. Nonetheless, elderly individuals are more likely to be affected by coronary artery diseases (CAD), and more than 80% of individuals who die from CAD are aged over 65^26^. Thus, the predicative value of the lipid profile may be higher in the elderly population. However, this hypothesis could not be confirmed by HDL-c levels in our cohort. The results from a meta-analysis also found that the risk of high total cholesterol to CAD mortality was attenuated in the subgroup analysis of older adults^27^.

HDL-c was once recognized as a potential target for therapeutic modification by many researchers^28,29^. Recent trials have focused their interest in effectively increasing HDL-c levels by administering niacin or cholesteryl ester transfer protein (CEPT) inhibitors. However, these therapies failed to improve clinical outcomes compared with placebo groups^8,9,11,12^. Moreover, in several contemporary studies, the protective effect of HDL-c was lost in certain patients, such as those with CAD or coronary artery bypass graft surgery^30–32^. Results from a pooled analysis of 6 community-based cohorts found elevated hazards of total mortality in the highest categories of HDL-c in men^15^. Moreover, recent evidence from large cohorts depicted a U-shaped association between HDL-c levels and mortality risk^33,34^. However, none of these studies focused on age-modifying effects on the relationship between HDL-c levels and all-cause mortality. In the present study, a very high level of HDL-c (≥80 mg/dl) was significantly associated with a high risk of all-cause mortality in individuals younger than 65. The association was independent of other cardiovascular factors and remained consistent in subgroup and sensitivity analyses. Thus, our finding strengthens the notion that increasing the level of HDL-c absolutely may not guarantee good prognosis, and the modification effect of age should be considered in HDL-c for mortality risk stratification.

Interestingly, though no significant association was found between HDL-c levels and total mortality in the elderly, a U-shaped association similar to the pattern in younger participants was revealed in older individuals stratified by CVD at baseline. Similarly, Ding *et al*. found that high (≥70 mg/dl) levels of HDL-c have a significantly higher risk of all-cause and CVD mortality than the reference level (HDL-c ranging from 40 to 49 mg/dl)^30^. These findings indicate that though HDL-c has apparent antiatherogenic effects, a very high level of HDL-c may not necessarily represent protective effects and a good prognosis in CVD patients.

We are unable to illuminate the exact mechanism by which very high levels of HDL-c (≥80 mg/dl) contribute to all-cause mortality in individuals younger than 65. Genetic variance should be taken into consideration, as several studies using Mendelian randomization cast doubts on the causative role of HDL-c in protecting against CVD^16,35,36^. Furthermore, the association between HDL-c levels and mortality may be mediated through closely intertwined relationships with other risk factors. As seen in our cohort, individuals with high HDL-c are more likely to have increased alcohol consumption, high total cholesterol levels, and high blood pressure. Paunio *et al*. once suggested that increased alcohol intake may confound the association between HDL-c and mortality in men^37^. However, in our study, high levels of HDL-c was consistently associated with high total mortality risk after adjusting for alcohol use.

Some limitations of our study should be noted. First, we were unable to investigate the association between HDL-c levels and cause-specific mortality due to a lack of specific death certificate reports. Second, our study focused on the level of HDL-c because it is routinely tested in primary care. The other important aspects of HDL-c, such as the particle sizes or function of HDL-c, were not available at the population level. Third, the present participants included an occupational population in northern China with more men than women. Therefore, the results should be treated with caution when extrapolated to other populations. Despite these limitations, our study has apparent strengths, such as a large sample size with enough power to conduct subgroup analyses, a relatively long follow-up time, an extensive range of HDL-c levels, and the wide range of detailed covariate data.

## Conclusion

Our results, based on a large cohort in northern China, found that young individuals with very high HDL-c levels may have increased all-cause mortality risk and that elderly participants also may not benefit from a high level of HDL-c. Future research is warranted to confirm these findings and further investigate the underlying mechanisms.

## Supplementary information

Supplementary information

## Data Availability

The datasets generated and analyzed during the current study are available from the corresponding author upon reasonable request.
