# Supplementary material for: Association between high-density lipoprotein cholesterol and all-cause mortality in the general population of northern China
Source: Sci Rep. 2019 Oct 8;9:14426. doi: 10.1038/s41598-019-50924-4 (PMC6783426; doi:10.1038/s41598-019-50924-4)
Supplement: Supplementary file 1 — Supplementary information [file 41598_2019_50924_MOESM1_ESM.pdf]

Association between high-density lipoprotein cholesterol and all-cause mortality in the general population of northern China

Author: Xintao Li[1], Bo Guan[1], Yanjun Wang[1], Gary Tse[2,3], Fuquan Zou[3], Bin Waleed Khalid[3], Yunlong Xia[3], Shouling Wu[4]*, Jianhui Sun[1]*.

Supplementary Figure 1. The Kaplan-Meier survival curves for all-cause mortality by HDL-c levels.

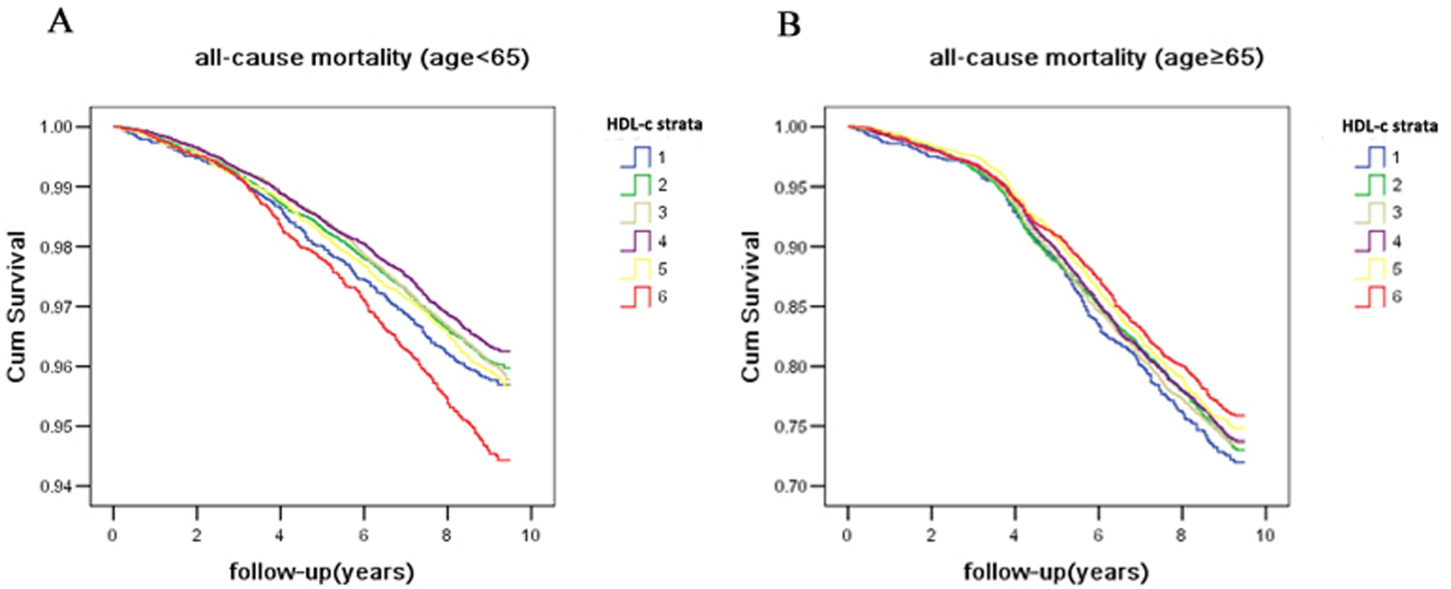
A
all-cause mortality (age<65)
Cum Survival
1.00
0.99
0.98
0.97
0.96
0.95
0.94
0
2
4
6
8
10
follow-up(years)
HDL-c strata
1
2
3
4
5
6
B
all-cause mortality (age≥65)
Cum Survival
1.00
0.95
0.90
0.85
0.80
0.75
0.70
0
2
4
6
8
10
follow-up(years)
HDL-c strata
1
2
3
4
5
6
